# Editorial: Cardioprotection, sex and gender differences

**DOI:** 10.3389/fphys.2022.940058

**Published:** 2022-08-31

**Authors:** Valentina Mercurio, Nina Kaludercic, Nazareno Paolocci, Claudia Penna

**Affiliations:** ^1^ Department of Translational Medical Sciences, Federico II University, Naples, Italy; ^2^ Department of Clinical and Biological Sciences, University of Turin, Turin, Italy; ^3^ Division of Cardiology, Department of Medicine, Johns Hopkins University School of Medicine, Baltimore, MD, United States

**Keywords:** gender, heart, estrogen, cardioprotection, COVID–19

The special issue, “Cardioprotection, sex and gender differences” focuses on various aspects of sex and gender and supports that they play a significant role in cardiovascular diseases (CVD). It has long been known, and supported by numerous studies, that sex differences play a major role in cardiac susceptibility to cardiovascular disease. Indeed, important and relevant disparities in pathophysiology, clinical presentation and management were observed between men and women. o date, the numerous molecular mechanisms underlying these differences are currently still partially unknown. It is important to underline the distinction between the two terms, sex and gender. While “sex differences” are merely due to biological differences, “gender differences” depend on many aspects, including the environment, lifestyle and characteristics of attitude. The use of experimental models and a careful analysis of clinical data is currently emerging that both disparities show fundamental importance both in the diagnosis and management of cardiovascular diseases. Therefore, gender differences may be considered a fundamental branch of precision medicine.

In the present special issue, these topics have been covered with both original works and reviews (Akther et al.; Leutner et al., 2021; Li et al.; Liu et al.; Xu et al.; Ytrehus et al., 2021; Yu et al., 2022). Forrny et al. analyzed these differences in the setting of the metabolic syndrome. The Authors focused their attention on type 2 diabetes, a chronic disease associated with micro and macrovascular complications. Indeed, the Authors reviewed the literature and reported an increased risk of CVD in women with diabetes compared to men, in particular concerning the risk of coronary heart disease accompanied by higher mortality in case of acute myocardial ischemia.

Another interesting aspect is reported by Querio et al. in a mini review on the response to cardioprotective maneuvers in different experimental models related to sex-dependent response. The Authors underline the influence of sex on the outcome of cardioprotective procedures. When applied, cardioprotection significantly reduces damage from ischemia/reperfusion. Within their review, Querio and collaborators highlighted that the protective maneuvers show effects that are not always positive when applied in female experimental models of a given age. The noticeable differences in response to these procedures are partly due to sex hormones, some of which decrease over the life span in women. The presence of sex hormones, in particular estrogen, has a highly protective role against ischemia/reperfusion damage. In this report Querio et al. describe the molecular pathways involved in cardioprotective protocols, clarifying at least in part how sex hormones can help improving physiological responses to CVD (Querio et al., 2021).

Ueda et al. discuss the significance of sex differences in the pathogenesis of cardiovascular disease. The Authors, through an overview of the results of the clinical studies obtained to date, relating to sex differences and hormone replacement therapy. The recent pandemic condition has highlighted important disparities in the pathogenesis of COVID, as reported by numerous studies and reviews (Pagliaro and Penna, 2020; Penna et al., 2020; Viveiros et al., 2021). In this regard, in relation to COVID19, in this special issue Cheng et al. found in a retrospective cohort study that the incidence of myocardial damage in patients with COVID-19 is sex-dependent, predominantly in association with a higher degree of inflammation and bleeding disorders in men. The paper reports the results of a retrospective study conducted on 1,157 COVID-19 patients (49.4% female and 50.6% male) who were hospitalized in Huoshenshan hospital from 12 March 2020 to 11 April 2020. The Authors emphasize the protective role played by sex hormones, in particular with regard to the inflammatory reaction and the state of coagulation. The latter, varying based on gender and women’s specific protective mechanisms, likely mediated by sex differences in the incidence of myocardial damage. Sex differences are maintained in the incidence of adverse outcomes in COVID-19 patients.

Another aspect related to a component of the COVID-19 scenario was presented by (Yu et al., 2022). The Authors analyzed the role of the angiotensin converting enzyme 2 (ACE 2) in the hypertensive heart. The results obtained indicate the presence of a male preponderance for an increase in the gene expression of ACE and ACE2. The results are in agreement with the role of androgens or male chromosomal complement in controlling the expression of the two ACE genes.

In conclusion, the studies published in this special issue confirm the importance of hormonal balance in determining CVD, an aspect that has also been apparent during the COVID-19 pandemic.
